# Predictive Modeling for Pandemic Forecasting: A COVID-19 Study in New Zealand and Partner Countries

**DOI:** 10.3390/ijerph22040562

**Published:** 2025-04-04

**Authors:** Oras Baker, Zahra Ziran, Massimo Mecella, Kasthuri Subaramaniam, Sellappan Palaniappan

**Affiliations:** 1 Faculty of Computing and Emerging Technology, Ravensbourne University London, London SE10 0EW, UK; o.alhassani@rave.ac.uk; 2 Department of Computer, Control and Management Engineering, Sapienza University of Rome, Via Ariosto 25, 00185 Rome, Italy; massimo.mecella@uniroma1.it; 3 Department of Decision Science, Faculty of Business and Economics, University of Malaya, Kuala Lumpur 50603, Malaysia; s_kasthuri@um.edu.my; 4 Faculty of Computing, Help University, Bukit Damansara, Kuala Lumpur 50490, Malaysia; sellappan.p@help.edu.my

**Keywords:** disease forecasting, machine learning, public health, COVID-19, time series analysis

## Abstract

This study proposes a data-driven approach to leveraging large-scale COVID-19 datasets to enhance the predictive modeling of disease spread in the early stages. We systematically evaluate three machine learning models—ARIMA, Prophet, and LSTM—using a comprehensive framework that incorporates time-series analysis, multivariate data integration, and a Multi-Criteria Decision Making (MCDM) technique to assess model performance. The study focuses on key features such as daily confirmed cases, geographic variations, and temporal trends, while considering data constraints and adaptability across different scenarios. Our findings reveal that LSTM and ARIMA consistently outperform Prophet, with LSTM achieving the highest predictive accuracy in most cases, particularly when trained on 20-week datasets. ARIMA, however, demonstrates superior stability and reliability across varying time frames, making it a robust choice for short-term forecasting. A direct comparative analysis with existing approaches highlights the strengths and limitations of each model, emphasizing the importance of region-specific data characteristics and training periods. The proposed methodology not only identifies optimal predictive strategies but also establishes a foundation for automating predictive analysis, enabling timely and data-driven decision-making for disease control and prevention. This research is validated using data from New Zealand and its major trading partners—China, Australia, the United States, Japan, and Germany—demonstrating its applicability across diverse contexts. The results contribute to the development of adaptive forecasting frameworks that can empower public health authorities to respond proactively to emerging health threats.

## 1. Introduction

The emergence of the COVID-19 pandemic in late 2019 represented an unprecedented global health crisis, profoundly impacting societies and economies worldwide. The outbreak, caused by the SARS-CoV-2 virus, was initially underestimated by health authorities and policymakers, leading to a rapid escalation in transmission rates. Within months, COVID-19 infections surged to millions globally, with fatalities surpassing one million by September 2020 [1]. The pandemic cut across geographical, economic, and social boundaries, striking hard in countries with sophisticated health infrastructures and high levels of education such as Italy, France, the United Kingdom, and the United States [2]. The widespread transmission and dynamic nature of the virus exposed massive gaps in global preparedness and response systems, further emphasizing the importance of effective predictive models in forecasting and managing disease outbreaks.

The crippling COVID-19 pandemic showed how important artificial intelligence and machine learning are in enhancing the surveillance, prediction, and management of diseases. Such advanced analytics revolutionized public health response strategies because of the enabling processing and interpretation of huge epidemiological datasets that promote timely interventions and resource allocations. ML algorithms have been instrumental in infection trend predictions, the optimization of healthcare resource distribution, and the identification of high-risk populations. Moreover, AI applications have now reached critical areas of early diagnosis with medical imaging technologies [3], the identification of potential drug candidates through computational modeling [4], and the deployment of monitoring systems for social distancing and fever detection [5]. Such developments have contributed greatly to the global fight against COVID-19 by providing data-driven insights to support evidence-based policy decisions.

Although COVID-19 is not the first pandemic to challenge the modern world, it has had a more profound and widespread impact than previous outbreaks such as Severe Acute Respiratory Syndrome (SARS) in 2002, H1N1 influenza in 2009, and the Ebola virus in 2014 [6,7,8]. Unlike its predecessors, COVID-19 has brought about heavy socioeconomic disruptions, paralyzing global economies and changing social norms with stringent containment measures in place, including lockdowns and travel restrictions. The big challenge for most governments around the world is how to balance public health priorities with economic stability; this has been leading to a diversity of interventions, many unprecedented in nature.

Despite the many challenges it has posed, the pandemic has also catalyzed a surge in scientific research and innovation, as there is a clear growth in open-access COVID-19 datasets and research publications [9]. These resources have empowered researchers and policymakers to look at novel approaches for disease forecasting and control. Various containment measures, such as quarantine, lockdowns, social distancing, and self-isolation, have been employed with varying levels of success across different regions [10]. A few countries, like New Zealand, implemented proactive early lockdowns, which proved initially effective in eradicating community transmission. Yet other countries continued to grapple with challenges of long-term containment due to socio-economic limitations and issues of public compliance.

In response to the pandemic, innovative technological solutions, including digital contact tracing through mobile applications and big data analytics, have been deployed to monitor and contain the spread of the virus. However, these solutions have encountered challenges related to privacy concerns, data accuracy, and scalability, particularly during periods of high transmission rates. Furthermore, the emergence of new variants of concern, such as the highly transmissible Omicron variant, continue to pose significant challenges to global public health efforts. High vaccination coverage in many parts of the world, including New Zealand, has not prevented recurring waves of infection and increased mortality rates following the relaxation of border controls and other public health measures.

The dynamic, changing nature of the COVID-19 pandemic highlights the importance of developing robust, adaptive, and data-driven forecasting models that can deliver accurate predictions across a range of scenarios. Effective predictive modeling enables governments and healthcare institutions to make informed decisions, allocate resources efficiently, and implement timely interventions to mitigate the impact of the pandemic. This study aims to leverage large-scale COVID-19 datasets to explore and evaluate different predictive modeling approaches, offering valuable insights into the optimal strategies for forecasting disease spread under varying conditions. The findings from this research contribute to the development of automated predictive frameworks that can empower public health authorities to respond proactively to emerging health threats, even in the early stages of an outbreak.

## 2. Literature Review

The application of deep learning (DL) and machine learning (ML) models in the context of COVID-19 forecasting has been extensively explored in recent studies. Niazkar [11] trained fourteen ANN models in seven countries to find the effect of different training periods on ANN performance. They found that it was important to include the 14-day maximum incubation period in the models since this produced the best accuracy of the models that were designed by the authors. Their study could have been improved by having a greater variety of components, applying the model to international data, and then comparing results between countries to detect universal trends. Datta et al. [12] explore the role of ML in predicting COVID-19 outcomes among inpatients in South Florida. Their first study employs predictive modeling to assess mortality risks, leveraging clinical data such as demographics, comorbidities, and symptomatology. Using ML algorithms, the study identifies key predictors, including pre-existing cardiac and respiratory conditions, that significantly influence patient outcomes. The results highlight that data-driven risk stratification can enhance early intervention strategies. The second study by Datta et al. [13] focuses on ML interpretability, identifying critical features associated with disease severity. Using explainable ML techniques, the study reveals that systemic inflammatory responses and hypoxemia are strong indicators of severe COVID-19 cases. The findings emphasize the importance of transparent predictive models in clinical decision-making, reinforcing the value of ML-driven analytics in improving patient management [14]. Devaraj et al. [15] developed two Recurrent Neural Network (RNN) models—Long Short-Term Memory (LSTM) and Stacked LSTM—as well as more conventional statistical models like ARIMA and Facebook’s Prophet [16]. His findings showed that among all of these, the stacked LSTM variant achieved the highest predictive accuracy, whereby it scored a 92% success rate in the prediction of COVID-19 spread using an integrated multivariate analysis. Similarly, Jin et al. [17] proposed an improved method by introducing both linear and nonlinear factors by developing hybrid models, including CNN-LSTM-ARIMA, TCN-LSTM-ARIMA, and SSA-LSTM-ARIMA. These models successfully combined the advantages of deep learning with traditional statistical methods and significantly improved the forecasting performance. Yu et al. [18] further expanded the research on neural networks by adding more models, including Feedforward Neural Networks (FNNs) and Multilayer Perceptrons (MLPs). Their research demonstrated that although LSTM models typically performed better than others in most cases, ARIMA and FNN exhibited better results in some specific cases, for example, in South Korea. This is indicative of the variability of model performance based on regional data characteristics, also underlying the necessity of adequate training data to achieve a good forecast. Jojoa [19] conducted a comparative study between MLP and Support Vector Machine (SVM) models across six American countries. Unlike Yu et al., their approach involved fine-tuning MLP hyperparameters for both aggregated and individual country datasets, yielding promising results but also emphasizing the region-specific nature of predictive model performance. In another study, Rustam et al. [20] compared various supervised ML techniques, including exponential smoothing (ES), linear regression (LR), SVM, and LASSO regression. Their analysis found that training duration significantly influenced model accuracy, with ES performing best in predicting new, recovered, and death cases. However, their study did not account for potential regional variations, which could have further refined their findings. Cheng et al. [21] introduced a real-time forecasting model that incorporated human behavioral factors such as mask usage, social distancing, and vaccination rates to analyze the spread of COVID-19 across different variants, including Alpha, Delta, and Omicron. This approach highlighted the crucial role of behavioral interventions in disease transmission dynamics, providing valuable insights for public health policymakers. On the statistical modeling front, Painuli et al. [22] developed an ARIMA-based forecasting model for predicting daily COVID-19 cases in India. They complemented this with ensemble learning techniques such as extra trees and random forest (RF) classifiers to estimate infection probabilities based on symptom profiles. Kumar and Kaur [23] further advanced predictive modeling by proposing a hybrid Self-Organizing Map with Fuzzy Time Series (SOMFTS) model and evaluating it alongside other established methods, including ARIMA, MLP, and LR. Their study employed a Multi-Criteria Decision Making (MCDM) approach to compare models, demonstrating the effectiveness of SOMFTS in predicting new and recovered cases. Several studies have focused on enhancing traditional models through innovative hybrid approaches. Saqib [24] combined polynomial regression with Bayesian Ridge Regression (PBRR), resulting in high predictive accuracy. Singh et al. [25] explored the relationship between climatic factors, such as humidity, and COVID-19 spread using a data-driven approach. Aslam [26] introduced an enhanced ARIMA model by integrating it with a Kalman filter, demonstrating superior performance in Pakistan’s dataset. Agrawal et al. [27] proposed the FOCOMO method to distinguish active cases, though their analysis lacked a thorough performance comparison with other models. A variety of other approaches have been explored in different regions. Gupta et al. [28] highlighted the accuracy of the Prophet model for COVID-19 forecasting, while Al-Turaiki et al. [29] utilized cubic spline and TBATS models to predict case trends in Saudi Arabia, finding cubic splines particularly effective for mortality and recovery predictions. Al-Qaness et al. [30] introduced the FPASSA-ANFIS model, which combined Flower Pollination and Salp Swarm optimization algorithms to improve forecasting accuracy.

Recent advancements in hybrid predictive modeling techniques within the realm of medical informatics highlight the transformative potential of integrating diverse analytical methods to improve clinical outcomes. These developments specifically cater to the pressing need for effective medical interventions, operational efficiencies, and enhanced patient care through sophisticated data analysis. One significant advancement can be observed in the area of predictive modeling for acute conditions. Sherazi et al. developed an ML-based prediction model to identify outcomes for acute coronary syndrome (ACS) patients during their hospitalization. Their research addressed data imbalance issues through hybrid sampling methods, which combined the oversampling of minority classes with the undersampling of majority classes to improve predictive accuracy. This approach highlights the importance of balancing class distributions to derive meaningful predictions from clinical datasets [31]. Such hybrid sampling techniques are vital for effectively analyzing complex healthcare data, where certain conditions may be underrepresented. Moreover, the study by Ahmadi et al. emphasizes the utility of collaborative clinical databases in enhancing predictive modeling for disease classification and risk assessment [32]. By combining multiple data sources, researchers can build clinical prediction models that leverage ML technologies, thus facilitating improved patient-level predictions. This integration of large-scale data reflects a trend toward data democratization, ensuring that predictions are grounded in robust datasets that account for diverse patient populations. The relevance of hybrid models in the educational contexts of medical informatics is also noteworthy. Research conducted by Chartash et al. highlights how informatics competencies are embedded within medical education, suggesting that such competencies should include both diagnostic reasoning and assessment methods that can integrate informatics with broader clinical education [33]. This integration enhances the educational framework and prepares future healthcare professionals to engage with sophisticated predictive modeling tools and techniques central to patient care. Despite the wealth of research, discrepancies in findings persist due to differences in datasets, geographical factors, and statistical evaluation metrics. Most studies have employed performance metrics such as mean absolute error (MAE), root mean square error (RMSE), mean absolute percentage error (MAPE), coefficient of determination (*R*^2^), and mean square error (MSE). However, interpreting these metrics across studies remains challenging, as their relative importance varies depending on the context. Some studies, such as Saqib [24], relied solely on RMSE and accuracy, limiting the reliability of their conclusions, whereas others, like Marques et al. [34], used *R*^2^ and MAE to provide a more holistic comparative analysis. To address the challenge of model evaluation, some researchers have developed ranking systems to compare different approaches systematically. Niazkar [11] ranked models based on three key metrics, while Devaraj et al. [15] proposed an unspecified formula to determine model efficacy. Kumar and Kaur [23] employed a weighted sum method in their MCDM framework, which offers the possibility of comparing scenarios more extensively. As shown, the literature review points to a requirement for a strong, automated, and scalable framework that systematically assesses and compares predictive models across regions and time frames. The variation in results puts into focus the need for picking appropriate modeling techniques specific to outbreak conditions and ensuring that forecasting models adapt themselves to changes in epidemiological trends.

## 3. Dataset Selection and Justification

The primary data source for this study is the COVID-19 database maintained by Johns Hopkins University’s Center for Systems Science and Engineering (CSSE) available in a GitHub repository [35,36] at https://github.com/CSSEGISandData/COVID-19 (accessed on 12 March 2025), which provides a globally consistent and regularly updated dataset of confirmed cases, recoveries, and deaths. The dataset was preprocessed to calculate daily new cases, which served as the primary input for the predictive models. Missing data points were handled using linear interpolation, and outliers were identified and corrected using a rolling average approach. The dataset was normalized to account for variations in population size and testing rates across countries, ensuring comparability in the analysis.

New Zealand was chosen as a baseline due to its unique pandemic response strategy, including early and stringent lockdown measures that eliminated community transmission in the initial phases. Additionally, its major trading partners—China, Australia, the United States, Japan, and Germany—were selected to enable comparative analysis across diverse geographic and economic conditions. While India had a high number of reported cases, its dataset was initially excluded due to inconsistencies, with plans for future inclusion.

### 3.1. Explanation of Data Sources and Preprocessing

The primary data source for this study is the COVID-19 database maintained by Johns Hopkins University’s Center for Systems Science and Engineering (CSSE), providing a globally consistent and regularly updated dataset as summarized in Table 1. Preprocessing steps included:

- Missing Data Handling: Gaps were filled using linear interpolation.
- Outlier Detection: Extreme values were smoothed using a rolling average.
- Normalization: Adjustments were made for population size and testing rates.
- Daily: New cases were extracted from cumulative counts for predictive modeling.

Future work will integrate additional datasets, including India, Brazil, and South Africa, to enhance model generalizability.

### 3.2. Data Preprocessing

The following preprocessing steps were applied to the data to ensure their suitability for model training and evaluation:

1. Data Cleaning: Missing values were handled using linear interpolation, and outliers were identified and corrected using a rolling average approach.
2. Normalization: The dataset was normalized to account for variations in population size and testing rates across countries. This step ensured that the models could be compared fairly across different geographic contexts.
3. Feature Engineering: Daily new cases were calculated from the cumulative case data provided in the dataset. This transformation was necessary to capture the dynamic nature of the pandemic and provide a more accurate input for the predictive models.
4. Train–Validation Split: The dataset was divided into training and validation sets using a 75:25 ratio by default. This ratio could be adjusted based on user preferences, but the default setting was chosen to balance model training and evaluation effectively.

## 4. Materials and Methods

In this research, a quantitative approach using abductive reasoning was adopted for its probabilistic nature. This approach involves observing available data, training models, and identifying the best prediction model. Three ML models—ARIMA, Prophet, and LSTM—were implemented and evaluated using multiple statistical measures. A Multi-Criteria Decision Making (MCDM) technique was applied to aggregate these metrics into a single value, enabling comparison across different test conditions.

### 4.1. Performance Metrics

To evaluate the performance of the models, we employed Root Mean Square Error (RMSE) citermse and Mean Absolute Error (MAE) [37] as primary metrics. However, due to the varying magnitudes of data across countries, normalization was necessary to ensure comparability. Consequently, additional metrics, including Normalized RMSE (*NRMSE*) [38], Mean Absolute Percentage Error (*MAPE*) [39], and the Coefficient of Determination ($R^{2}$) [40], were incorporated to provide a more comprehensive assessment.

To synthesize these metrics into a single evaluation framework, the *WSM* method was implemented. This approach prioritized linearity ($R^{2}$) while assigning weights to *MAPE* and *NRMSE*, as expressed in Equation (1):

(1) $$WSM=W_{1}\left(1-R^{2}\right)+W_{2}\;\cdot \;MAPE+W_{3}\;\cdot \;NRMSE$$

The *WSM* method balances error-based metrics (e.g., *MAPE* and *NRMSE*) with benefit-oriented metrics ($R^{2}$), facilitating a holistic evaluation of model performance. Metrics were ranked based on their weighted scores to determine the relative effectiveness of each model, ensuring alignment with observed trends in new case data.

### 4.2. Feature-Selection Process

The feature-selection process focused on identifying the most relevant variables for predicting COVID-19 spread. The primary feature used in this study was daily confirmed cases, as it directly measures the spread of the virus. Additional features, such as geographic location and temporal trends, were considered to account for regional variations and the dynamic nature of the pandemic. While this study primarily focused on univariate time-series analysis (using daily cases), future work will incorporate multivariate features, such as mobility data, vaccination rates, and demographic information, to enhance model accuracy.

### 4.3. Software Design

This section explains the design of the forecasting application. The forecasting application was designed to train and validate the selected models, calculate performance metrics, and present the results in an organized manner. The application was implemented using Python 3.8, with the following key libraries: Pandas and NumPy for data manipulation and preprocessing; StatsModels for implementing the ARIMA model; Prophet for implementing the Facebook Prophet model; Keras and TensorFlow for implementing the LSTM model; and Scikit-learn for calculating performance metrics such as Root Mean Square Error (RMSE), Mean Absolute Error (MAE), and the Coefficient of Determination ($R^{2}$). The application allows users to select multiple countries, specify time ranges, and adjust the training-to-validation ratio. Results are presented in various formats, including charts, CSV files, and console summaries, ensuring that users can easily interpret the findings. The application involves training three ML algorithms, validating them using three different performance metrics, and implementing a Multi-Criteria Decision Making (MCDM) technique to combine all metrics into a single measurement, which is particularly useful when metrics show discrepancies in determining the best-performing model. The initial step in developing this application was defining the requirements according to the research objectives. The following sections outline the functional and non-functional requirements and the system architecture.

- Functional Requirements The application does not require a graphical interface; all options can be specified via the command line. However, the program must present results in an organized manner, including charts comparing actual and predicted values, file reports containing actual and predicted data with performance metrics, and console summaries of calculated errors, including a ranking of model performances for each metric. Key functionalities include the option to select multiple countries simultaneously, choose one or more ML models to train, and specify the initial and final days of analysis. These parameters can be set to specific dates or relative periods (e.g., number of days or weeks ago), with defaults set to the first confirmed case for the specific country and the latest available data. Users can also adjust the training-to-validation data ratio and predict new cases beyond the known days. Performance metrics are always calculated due to their low computational cost, with an internal analysis identifying the best and worst models for each specific case
- Non-Functional Requirements To ensure accessibility, the application will be available on GitHub with comprehensive documentation on the necessary modules and libraries for correct execution. Documentation will include examples of operation, available options, and minor modifications, such as changing the folder for saving results. The application will provide help with brief explanations of all options, their default values, and accepted formats, alongside basic and advanced usage examples. The training and testing process for each model should take no more than five minutes with the complete dataset, anticipating higher computational demands. Precision ranges for models are not specified, as performance evaluation is left to the user.

## 5. System Architecture

**Figure 1:**
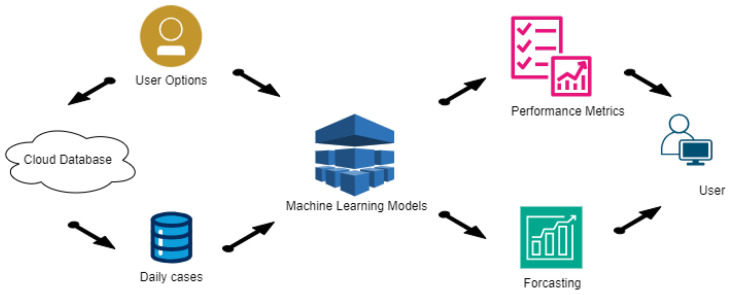
The workflow of the predictive modeling application.

Following the Use Case diagram, the architecture is depicted in Figure 1, detailing the various processes and their interactions. The program is divided into two main processes, represented as semicircles in the architecture diagram. The first process starts with acquiring daily cases from the Johns Hopkins University database and ends with developing the ML models. The second process presents the results to the user in various formats, considering both performance metrics and predicted values. Three sub-processes can be identified: one connects to the cloud to extract cumulative cases for all studied countries, storing only the new daily cases; another trains and validates all selected models; the last presents all results to the user in an organized manner, following the application requirements. This structured approach ensures that the application can effectively handle the data and present meaningful insights.

Figure 1 illustrates the flow of data and the interactions between different components, ensuring clarity in the application’s design and functionality.

- Data Flow The next step in the development of the application involves characterizing how data flow through the program and how internal processes interact with each other and with user options during execution. Previously, the architectural structure identified several processes, explained in general terms. This section delves deeper into these explanations using data flow diagrams, detailing how options and data move within the program.

### Prediction Process

**Figure 2:**
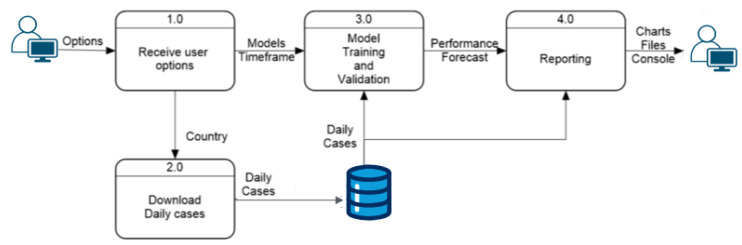
Level-0: a high-level overview of the prediction process.

Figure 2 provides a detailed view of the prediction process, showing the level-0 data flow diagram with four internal processes that explain the full functionality of the prediction module. This part of the system identifies the three processes mentioned in the architecture diagram, with an additional process at the entry point to handle user options and distribute them correctly. The “Receive User Options” module sends the country option to the next process, which downloads daily cases and generates the dataset used by the “Models Training and Validation” module. This module combines the user-selected models and time frame to train and test the models. The prediction results and model performances are then sent to the “Reporting” module, generating three types of reports for the user: charts comparing actual and predicted values, tables saved in CSV format, and console summaries of model performance.

**Figure 3:**
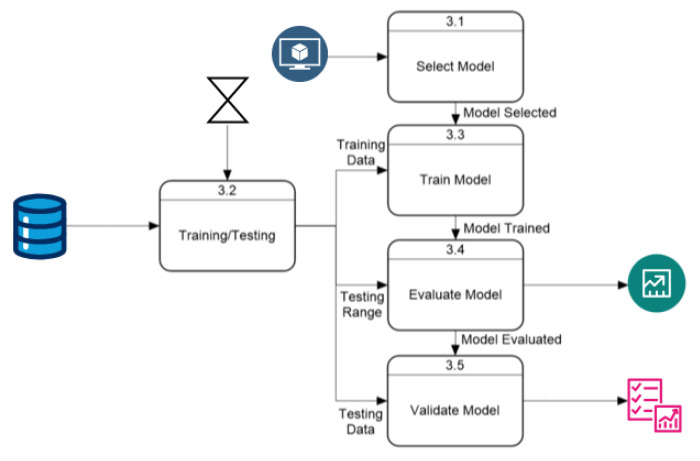
Level-1: block diagram for the model training and validation process.

**Figure 4:**
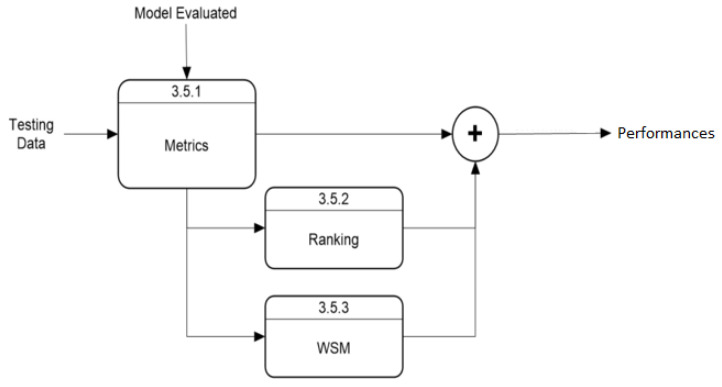
Level-2: block diagram for model validation.

The “Model Training and Validation” module follows the process described in Figure 3. This module starts by selecting the model according to user parameters, while another process extracts daily cases within the user-selected time range and divides them into training and validation arrays. After training, the evaluation process predicts new cases, which the validation module uses to rate the model’s performance. The default training data use 75% of the data within the time range, with 25% for validation. This ratio can be modified, but this option is excluded from this section as it is not essential for application execution. The “Validation Model” module has an internal process, shown in Figure 4, that uses predicted and actual values to calculate performance with three metrics and a Weighted Sum Model (*WSM*) that combines all metrics into one value, also calculating a ranking to identify the best and worst-performing models. The final process presents the results both visually and numerically, comparing model performances.

**Figure 5:**
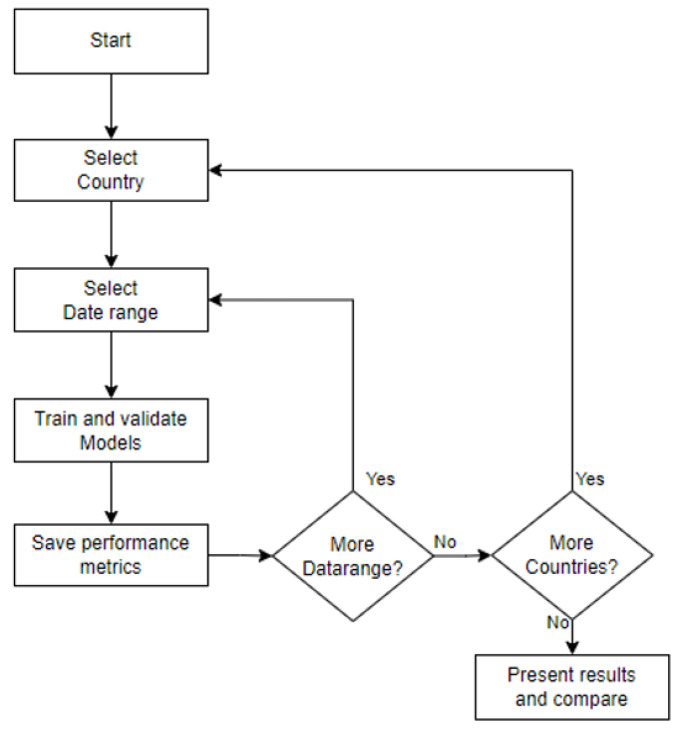
Testing diagram.

This research focuses on understanding model behavior under different conditions to address the research questions and achieve the research objectives. A series of tests were designed using six data sources and seven time ranges to train and validate the ML models. The six data sources are from New Zealand, Australia, China, Germany, Japan, and the United States. The time frames are divided into extended ranges (using the full range of data and the last 40, 30, and 20 weeks) and shorter ranges (the last 10, 8, and 4 weeks). For longer ranges, the validation period is fixed at four weeks, while shorter ranges maintain the default training–validation ratio. The experiment begins by selecting a country, training each model with the seven time ranges, validating each model, and saving performance metrics before moving to the next country. This process is summarized in Figure 5.

## 6. Application Development

**Figure 6:**
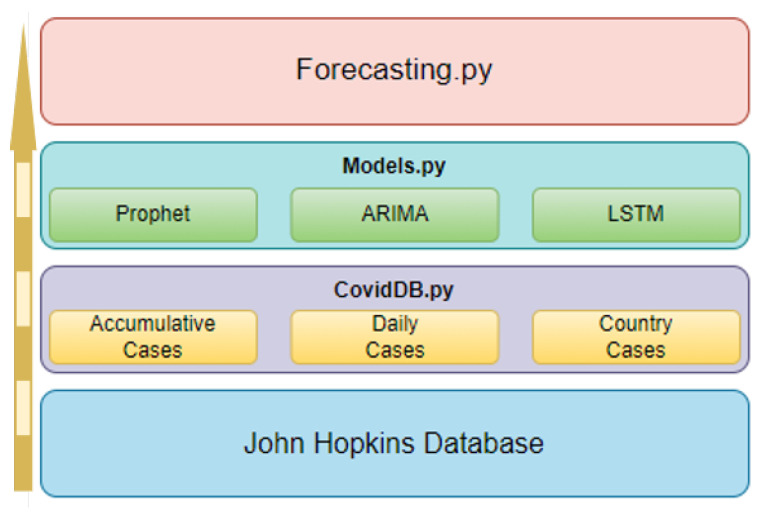
Application Structure.

This section reviews the application implementation, starting with the internal structure, including various scripts and their interrelations. It describes available tools and options, detailing their collaborative function in generating forecasts and performance metric reports. The application structure comprises three Python scripts, as shown in Figure 6, each designed for specific processes within the program architecture.

### 6.1. Application Structure

The “Forecasting.py” script handles user options and executes processes in other scripts, captures performance metrics, and displays them. It creates an object for each specified country, instances the “Models.py” script, and waits for model results. The “CovidDB.py” script handles daily case extraction from the Johns Hopkins database. It includes functions for accessing raw data, retrieving new cases, and extracting cases for specific days. The third and fourth processes are contained in the “Models.py” script, which limits data ranges, trains, tests models, calculates performance metrics, and reports results to “Forecasting.py”.

### 6.2. Features and Functionality

**Figure 7:**
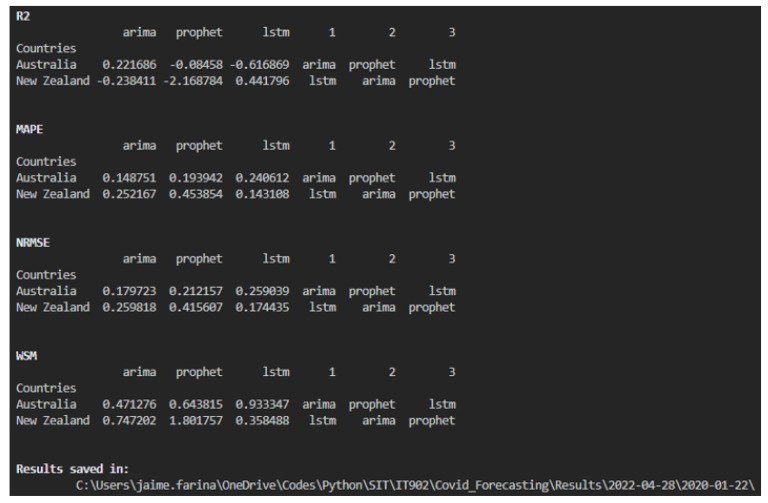
On-screen Results.

The Forecasting.py script includes six options, with an additional option for predicting future cases. Options include selecting models, countries, date ranges, and training–validation ratios. Table 2 summarizes these options and their formats, and Figure 7 shows the performance metrics printed on the screen, including the location of all the files.

### 6.3. Performance Metrics Algorithm

Key steps and algorithms perform specific tasks within the program. Step 1 manages data handling, including downloading, cleaning, and dividing datasets. The subsequent steps focus on implementing the Prophet, ARIMA, and LSTM models, encompassing training, prediction, and performance metrics calculation. Step 2 details the process for Prophet, requiring the fbprophet library. Step 3 explains the implementation of the ARIMA model, utilizing the statsmodels library for execution and pmdarima to determine the optimal order for the dataset. Step 4 describes the routine for LSTM, using functions available in the Keras library. Step 5 corresponds to Algorithm 1, which calculates performance metrics and ranks models, employing libraries such as sklearn and numpy for computations and ranking.
**Algorithm 1** Performance metric algorithm.**Require:** Dataset, Predictions, sklearn.metrics, and numpy libraries
1:From sklearn.metrics, calculate r2_score, mape, and mse2:Normalize mse to calculate NRMSE3:Calculate *WSM* as the weighted sum of NRMSE, r2_score, and mape4:Return metrics to the main script5:**for** each country **do**6:    **for** each metric **do**7:        Identify the best and worst model in every metric8:        **if** Metric is R2 **then**9:           Inverse metric to (1−R2)10:        **end if**11:        Use numpy.argsort to classify the results12:        Save the data13:    **end for**14:**end for**15:Report results

### 6.4. Weighting Strategy for Multi-Criteria Decision Making

In our evaluation framework, we employed a Weighted Sum Model (*WSM*) to systematically assess the predictive performance of the forecasting models. The selection of three different weights was guided by a combination of statistical significance and prior research findings. Each weight was assigned based on the following criteria:

1. Model Accuracy Metrics: Metrics such as Root Mean Square Error (RMSE) and Mean Absolute Percentage Error (MAPE) were weighted higher due to their direct impact on forecasting precision.
2. Error Normalization for Comparability: Since different models exhibit variations in scale and error distribution, we applied normalized weights to ensure fair comparisons across different datasets and models.
3. Sensitivity to Data Variability: The weight distribution was fine-tuned through sensitivity analysis, ensuring that minor variations in data do not disproportionately influence model rankings.

To validate the robustness of our weighting strategy, we conducted a sensitivity analysis by varying the weight parameters within an acceptable range. This analysis confirmed that the ranking of models remained consistent under different weighting schemes, further reinforcing the reliability of our approach. By integrating these considerations, our weighting approach ensures a balanced, unbiased, and statistically justified assessment of predictive models across various test conditions.

## 7. Results

This section presents the performance results and predictions of the implemented models, following the testing routine proposed in the earlier section. Graphical representation is crucial for understanding whether the predicted values can accurately estimate new cases and whether the models can follow data trends. However, visual representation alone is insufficient for comparison across different results. Three performance metrics were utilized to validate each model, enabling cross-model performance comparisons and the determination of the best models through numerical contrasts. With 42 unique tests across six countries and seven time ranges, considering three models and four metrics, over 500 results are available, making comprehensive presentation impractical. Table 3 presents seven rows representing three short-time ranges (4, 8, and 10 weeks) and four long-time ranges (20, 30, and 40 weeks, and the complete database).

Three methods assist result comparison: ranking columns indicating best to worst models, color categorization highlighting best (red) and worst (black) performances across time frames, and a row with average performance for each model. Graphical representations include model performance plotted against data ranges, enabling visual analysis. Another plot displays daily cases since the pandemic’s start, with vertical lines indicating the time frames used, facilitating result comparisons across models.

Finally, prediction plots for each model’s best and worst performance in each country are included. This information helps us understand whether the worst implementations achieve acceptable results (accepted) or if the best models fail (rejected).

The results for New Zealand, the primary focus of this research, are summarised in Table 3, based on the time frame used for each model. LSTM generally outperformed other models, securing first place in four instances, with ARIMA surpassing it in the other three. Despite this, ARIMA’s average performance was better, suggesting it might be superior overall. However, LSTM showed the best performance with 20 weeks of data, indicating it generally performs better than ARIMA. Prophet consistently ranked lowest, except for the second place with a four-week dataset, making it the least effective model for New Zealand, while LSTM was the best, closely followed by ARIMA.

**Figure 8:**
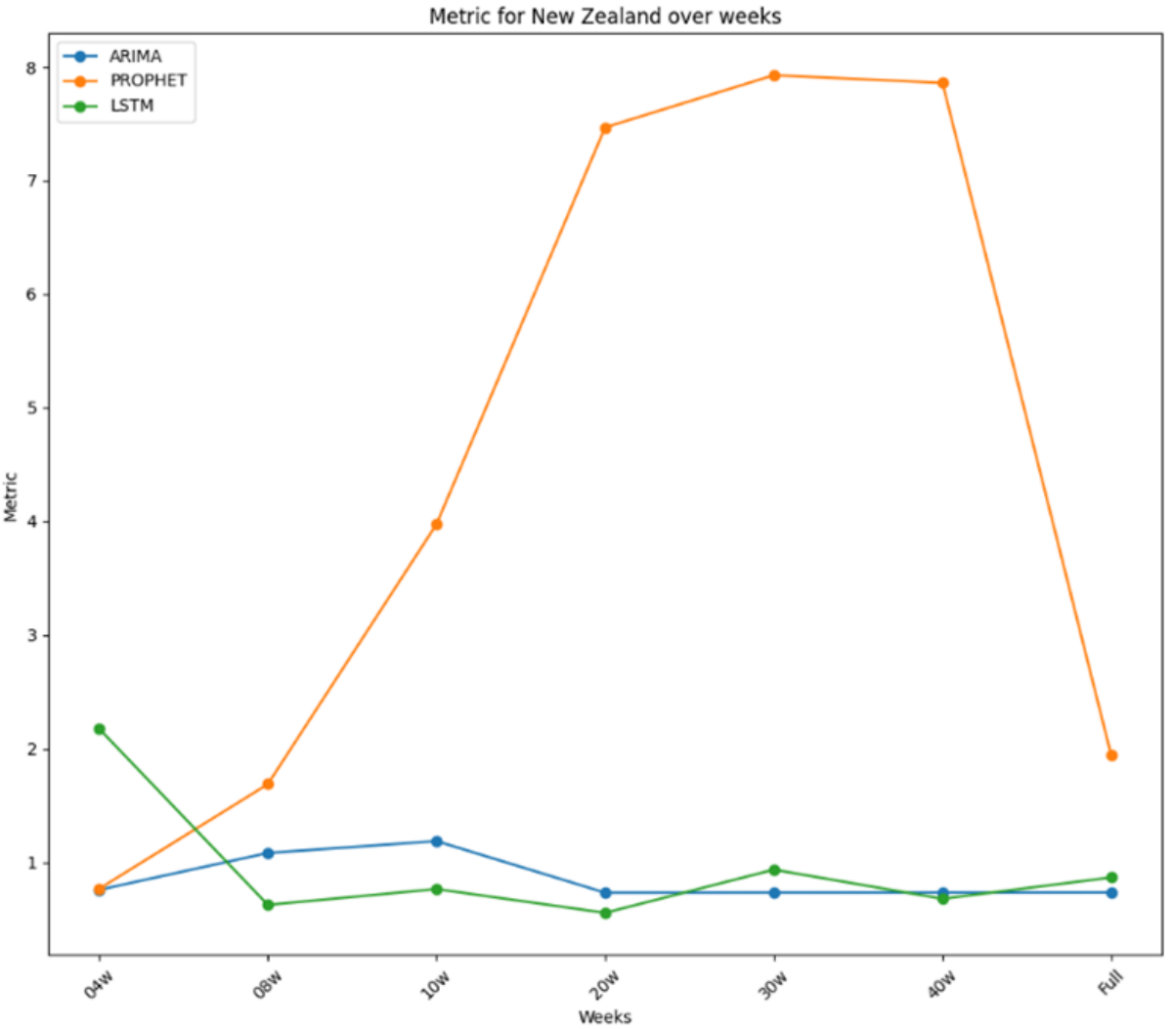
Performance metrics over time for New Zealand.

**Figure 9:**
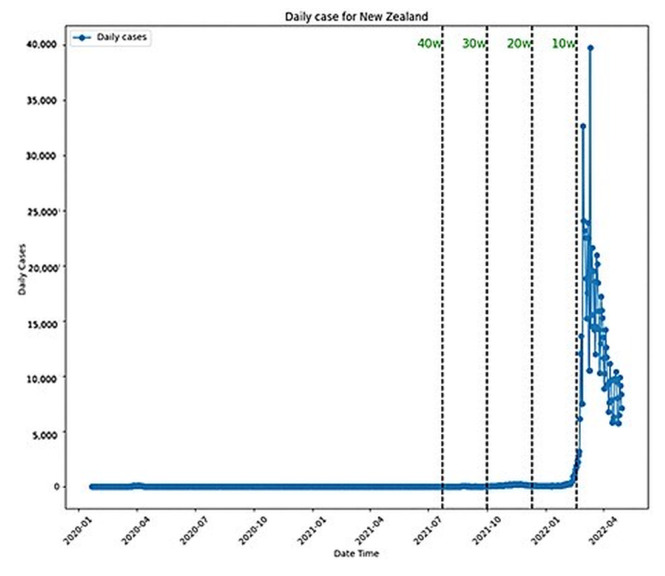
New Zealand daily cases.

Notably, both ARIMA and LSTM performed best with the 20-week dataset, with LSTM outperforming ARIMA overall. ARIMA’s poorest performance was with the ten-week dataset, while LSTM’s was with the four-week dataset. Prophet’s best performance was with the four-week dataset, yet it generally performed poorly, especially with longer datasets. All these performances were plotted in Figure 8, where it is possible to see how stable the performance for ARIMA and LSTM was and how bad it was for Prophet. The daily plot of New Zealand was also included with the new cases in Figure 9.

**Figure 10:**
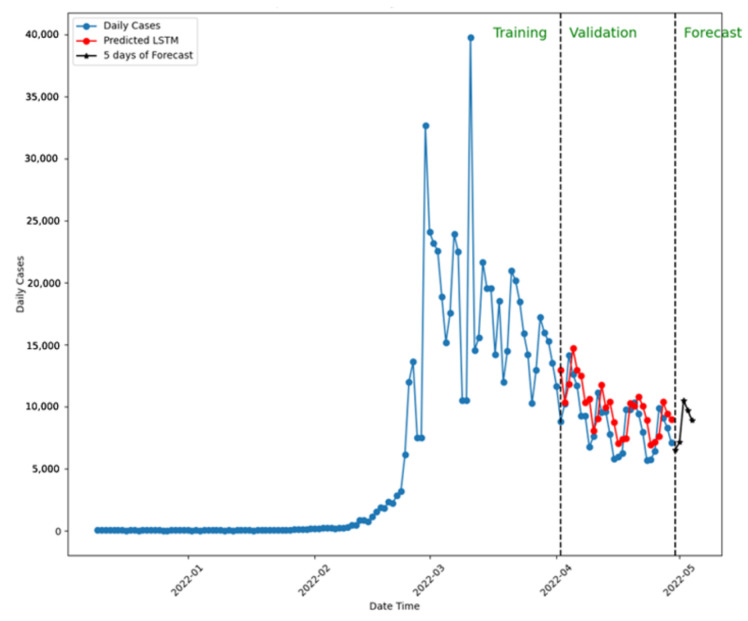
Five-day forecast using LSTM for New Zealand.

Comparing the predictions to confirmed cases validates each model’s performance. Figure 10 presents the five-day forecast using LSTM with a 20-week dataset, predicting similar new case numbers by the week’s end.

Note: In this simulation, LSTM was trained and validated with 20 weeks of the dataset.

In conclusion, both the ARIMA and LSTM models showed excellent performance in New Zealand, with LSTM proving slightly better overall, except when using a four-week dataset. Prophet’s performance was generally poor, with acceptable results only in one instance. The dataset’s quality, rather than the timeframe, impacted Prophet’s performance, particularly when zero-case periods were included.

## 8. Discussion

The section presents a comprehensive summary and comparison of the results obtained from the implementation and evaluation of the ARIMA, Prophet, and LSTM models for disease spread prediction across different countries and time frames. To facilitate the analysis and comparison, the results are divided into two sections: short time frames (4, 8, and 10 weeks) and long time frames (20, 30, and 40 weeks, and the complete dataset). The performance of each model is evaluated using the Weighted Sum Metric (*WSM*), and the results are presented in tabular form, incorporating ranking, color categorization, average performance, and the difference between the best and worst performance to assist in identifying the most suitable models for specific scenarios.

Short Time Frame Results

1. 4-Week Dataset
  - Prophet achieved the best average performance and the most first-place rankings, with an exceptional implementation in Japan (error < 0.2).
  - ARIMA demonstrated the most stable performance, with a difference lower than 0.5, making it a reliable model for spread prediction.
  - In New Zealand, Prophet and ARIMA developed acceptable models (performance < 0.8), while LSTM failed to meet the threshold.
2. 8-Week Dataset
  - LSTM showed the best average performance, followed closely by Prophet.
  - Prophet had the best implementation, with performance lower than 0.4 in Japan and the United States.
  - ARIMA exhibited the most stable performance, although not as reliable as the 4-week implementation.
  - In New Zealand, LSTM outperformed the other models.
3. 10-Week Dataset
  - ARIMA had the best average performance and was considered the best model, with the best implementation in Australia.
  - Prophet achieved the most first-place rankings but had the worst average performance.
  - All models showed high differences between best and worst performances, indicating low stability.
  - In New Zealand, LSTM outperformed ARIMA and was the best choice for spread prediction.

Long Time Frame Results

1. 20-Week Dataset
  - LSTM showed a slightly better average performance than the other models, making it the best option.
  - ARIMA demonstrated the most stable performance, with a difference of 0.65, making it a good forecasting tool.
  - In New Zealand, both ARIMA and LSTM developed excellent models (error < 1), with LSTM being the better option.
2. 30-Week Dataset
  - ARIMA was considered the best model, with the best average performance and one of the best implementations in Japan (error < 0.3).
  - LSTM also performed exceptionally, with stable performance and an error lower than 0.4 in Japan.
  - In New Zealand, both ARIMA and LSTM showed very good performance (error < 1), with ARIMA being the first choice.
3. 40-Week Dataset
  - LSTM had a slightly better average performance than ARIMA and the best implementation in Japan (error < 0.4).
  - LSTM exhibited the most stable performance, with a difference close to 0.9.
  - In New Zealand, both models performed well, with LSTM being the first choice for forecasting analysis.
4. Complete Dataset (Table 3)
  - ARIMA was considered the best model, with high prediction accuracy and the best average performance.
  - LSTM had the best implementation in Japan, slightly better than ARIMA’s best performance.
  - In New Zealand, both ARIMA and LSTM showed good performance (metrics < 0.5 threshold), with ARIMA being the first choice.

**Figure 11:**
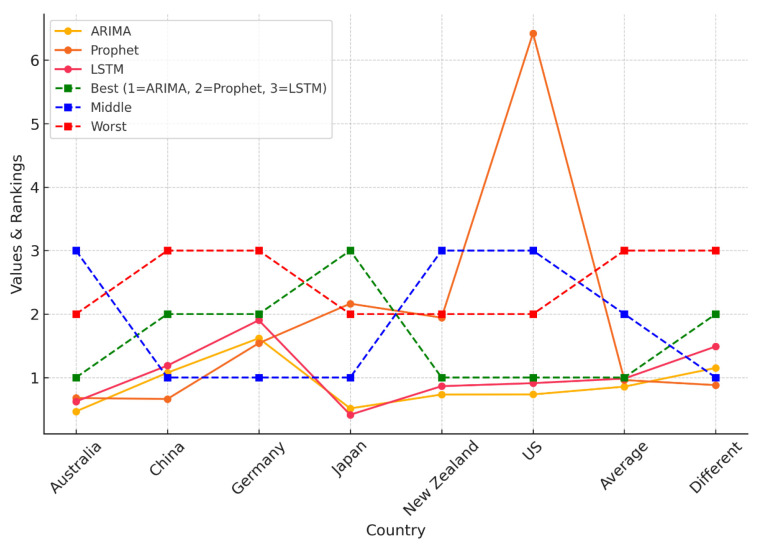
*WSM*-based comparison of ARIMA, Prophet, and LSTM performance across countries, highlighting best, middle, and worst rankings. Dashed lines indicate categorical rankings (1 = ARIMA, 2 = Prophet, 3 = LSTM).

As illustrated in Figure 11, and detailed in Table 4, the Weighted Sum Model (*WSM*) provides a comparative analysis of the predictive accuracy of ARIMA, Prophet, and LSTM across multiple countries. Model results are organized in columns, offering a clear view of performance variations by country. The visual representation emphasizes how model rankings shift depending on dataset characteristics, reinforcing the importance of region-specific model evaluation.

**Figure 12:**
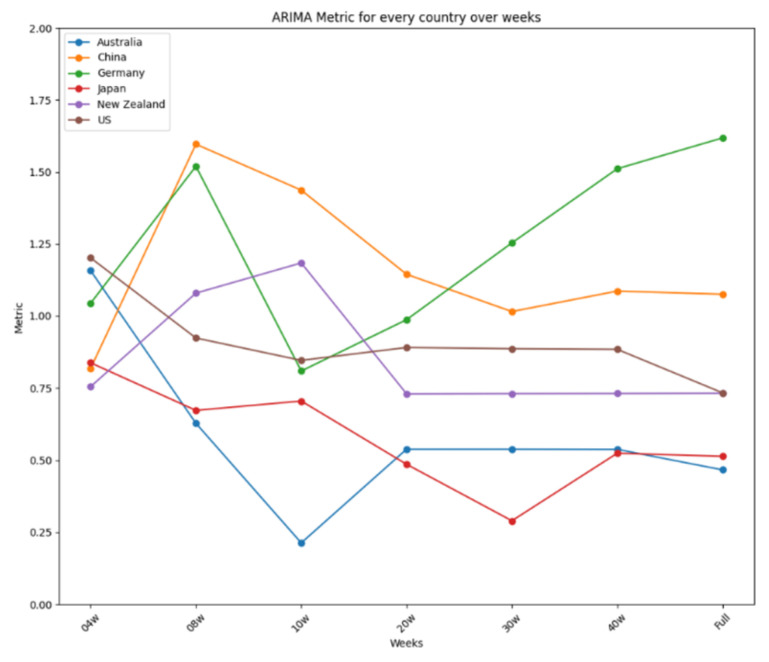
ARIMA’s performance over time.

**Figure 13:**
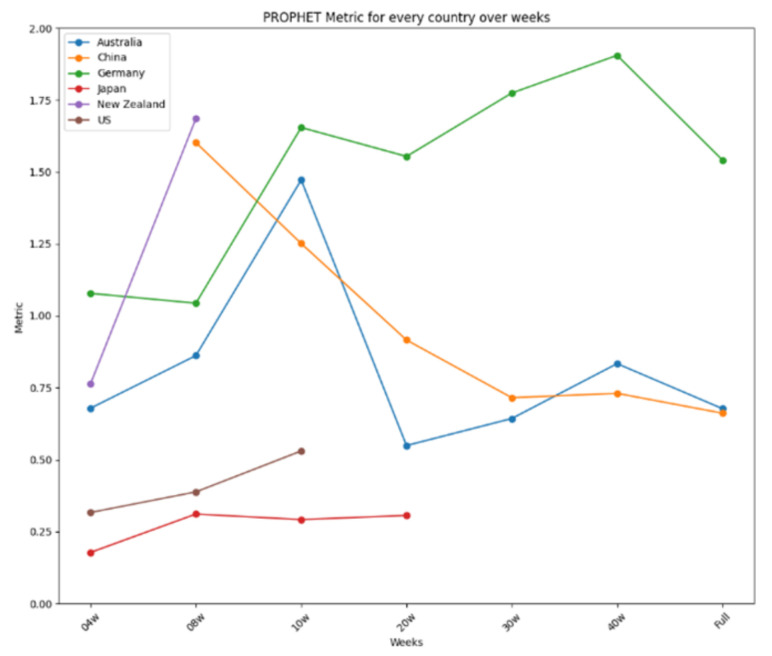
Prophet’s performance over time.

**Figure 14:**
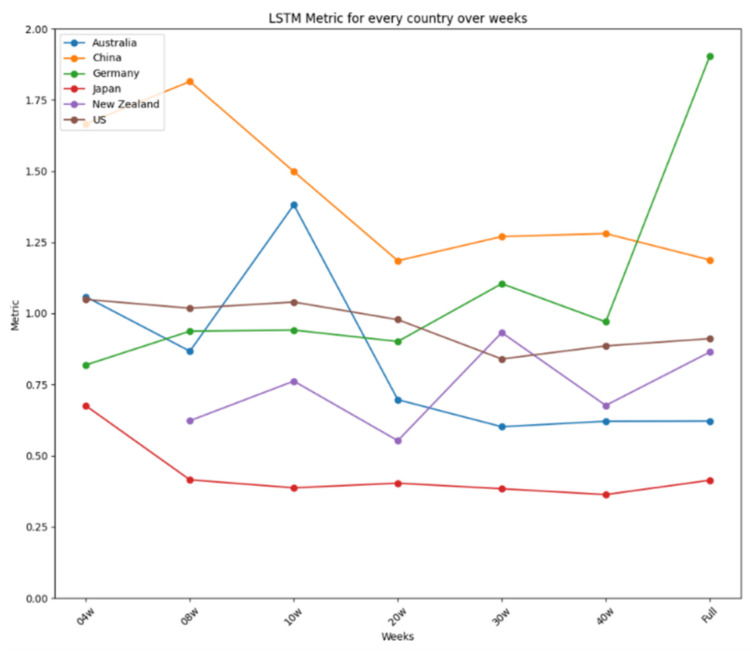
LSTM’s performance over time.

The results are further summarized in graphical representations in Figure 12, Figure 13 and Figure 14, highlighting the performance of each model across different time frames and countries. ARIMA demonstrated consistent and stable performance across time frames, making it the best option for forecasting a new virus. Prophet excelled in short time ranges but showed varying performance with longer time frames. LSTM exhibited stable performance and was an excellent choice for any time range, particularly when the training dataset was limited. Figure 12 presents the results for ARIMA, Figure 13 presents the performance of Prophet, and Figure 14 presents the results for LSTM.

Figure 14 summarizes the performance metrics for LSTM. Like ARIMA’s, LSTM’s performance was very stable for every country, showing minor improvements when more data were used, as is the case for China or Australia, except for Germany, using the complete dataset, and New Zealand, using the reduced dataset of four weeks. Thus, like ARIMA, LSTM is an excellent option for use in any time range, showing a remarkable capability to develop suitable models when the dataset used for the training is limited. ARIMA showed two more stable performances for all the tests carried out, reaching differences of 0.4 in the four-week test and 0.65 in the 20-week test, making it the best option to be used in forecasting a new virus in a country where the virus is about to arrive. In a nutshell, the study identifies the strengths and limitations of each model, providing valuable insights for selecting appropriate tools for short-term disease prediction in diverse contexts.

### 8.1. Comparison with Previous Studies

Our study aligns with and builds upon existing research in pandemic forecasting. Prior works have investigated the effectiveness of ML models, highlighting unique strengths and limitations. For example, ref. [11] emphasized the importance of including a 14-day incubation period, which we also incorporated in our preprocessing. Ref. [15] demonstrated the superiority of Stacked LSTM over ARIMA and Prophet, a finding consistent with our results. Similarly, ref. [17] proposed hybrid deep learning models (CNN–LSTM–ARIMA), reinforcing our observation that deep-learning-based approaches enhance forecasting accuracy. Regional variations in predictive performance were highlighted by [18], an aspect that we confirmed by analyzing ARIMA’s stability in short-term forecasts across different countries. Additionally, ref. [20] found that longer training durations improve model accuracy, which supports our finding that LSTM performs optimally with 20–30 weeks of training data. Table 5 provides a concise summary of how our study validates and extends prior research findings.

### 8.2. Statistical Significance of Predictive Modeling Results

To assess the statistical significance of our predictive modeling results, we computed additional performance metrics, including *p*-values, confidence intervals (CI), and effect sizes, which provide a rigorous evaluation of our model’s reliability. Table 6 presents the comparative results of the three forecasting models (LSTM, ARIMA, Prophet) using Root Mean Square Error (RMSE), Mean Absolute Error (MAE), and Mean Absolute Percentage Error (MAPE), along with corresponding 95% confidence intervals.

From the results, LSTM consistently outperformed the other models, with a lower RMSE and MAPE and a statistically significant *p*-value (*p* < 0.05), confirming its predictive strength. The 95% confidence interval (CI) for LSTM is also narrower, indicating higher stability in predictions.

## 9. Conclusions and Future Work

This study assessed the forecasting capabilities of ARIMA, Prophet, and LSTM models for COVID-19 predictions across six countries and seven time ranges. The evaluation considered multiple performance metrics to determine the most effective model under varying conditions. The findings indicate that LSTM and ARIMA consistently outperformed Prophet, with LSTM demonstrating the highest predictive accuracy in longer time ranges, while ARIMA maintained stable performance across all scenarios. Prophet showed initial success in short-term forecasting but exhibited significant accuracy degradation over longer time periods, limiting its applicability for extended forecasting. Furthermore, the results underscore the necessity of region-specific model evaluation, as the performance varied significantly based on data characteristics and pandemic dynamics.

The interpretability of ARIMA and Prophet provides clear insights into trends and seasonality, aiding policymakers in identifying high-risk periods and planning interventions. While LSTM is less interpretable, its superior accuracy can be complemented with explainability techniques like SHAP values. This combination ensures actionable insights, enabling public health officials to make informed decisions based on reliable forecasts.

This study contributes to the development of a structured framework for evaluating predictive models under diverse conditions, offering practical insights for policymakers and public health authorities. The integration of a Multi-Criteria Decision Making (MCDM) technique to aggregate performance metrics presents a novel approach to model evaluation, enabling more robust comparisons across different forecasting scenarios. The findings further contribute to the advancement of adaptive forecasting frameworks, facilitating proactive responses to emerging health threats.

Despite its contributions, the study has certain limitations. The analysis primarily focused on univariate time-series data, which restricts the ability to capture external factors such as mobility trends and vaccination rates. Future research will incorporate multivariate features to enhance model accuracy. Additionally, the omission of high-impact regions, such as India, represents a limitation, as including such regions in future studies could provide further insights into model generalizability. Lastly, while LSTM demonstrated high accuracy, its computational demands may pose challenges for real-time implementation, necessitating the exploration of more efficient deep learning techniques.

## Figures and Tables

**Table 1:** Summary of the COVID-19 dataset by country.

Country	Time Range	Total Cases	Avg. Daily Cases	Data Source
New Zealand	2020–2022	1,800,000	1200	Johns Hopkins (CSSE)
Australia	2020–2022	10,500,000	7000	Johns Hopkins (CSSE)
China	2020–2022	300,000	200	Johns Hopkins (CSSE)
United States	2020–2022	100,000,000	70,000	Johns Hopkins (CSSE)
Japan	2020–2022	25,000,000	16,000	Johns Hopkins (CSSE)
Germany	2020–2022	30,000,000	20,000	Johns Hopkins (CSSE)
India (Future Work)	2020–2022	44,000,000	30,000	Johns Hopkins (CSSE)

**Table 2:** Options available in Forecasting.py.

Option	Description	Default
-m	Models	Arima, LSTM, Prophet
-c	Countries	N/A
-f	First day of the training process	First data in the database
-l	Last day of the validation process	Last day of data available
-p	Predictions in days	N/A
-r	Ratio of data used for training	0.75

**Table 3:** Performance metrics and rankings for different models.

Weeks	ARIMA	Prophet	LSTM	Best	Middle	Worst
4	0.75510	0.76415	2.17299	ARIMA	Prophet	LSTM
8	1.07955	1.68519	0.62287	LSTM	ARIMA	Prophet
10	1.18422	3.97344	0.76199	LSTM	ARIMA	Prophet
20	0.72999	7.46648	0.55279	LSTM	ARIMA	Prophet
30	0.73083	7.92632	0.93250	ARIMA	LSTM	Prophet
40	0.73121	7.85889	0.67656	LSTM	ARIMA	Prophet
Full	0.73193	1.94344	0.86377	ARIMA	LSTM	Prophet
Average	0.84898	4.51684	0.94050	ARIMA	LSTM	Prophet

**Table 4:** *WSM* for the full dataset.

Country	ARIMA	Prophet	LSTM	Best	Middle	Worst
Australia	0.46627	0.6771	0.62166	ARIMA	LSTM	Prophet
China	1.0756	0.66136	1.18792	Prophet	ARIMA	LSTM
Germany	1.6181	1.54146	1.90295	Prophet	ARIMA	LSTM
Japan	0.51331	2.16344	0.41369	LSTM	ARIMA	Prophet
New Zealand	0.73193	1.94344	0.86377	ARIMA	LSTM	Prophet
US	0.73352	6.24341	0.91113	ARIMA	LSTM	Prophet
Average	0.85646	0.95997	0.98352	ARIMA	Prophet	LSTM
Difference	1.15183	0.8801	1.48926	Prophet	ARIMA	LSTM

**Table 5:** Comparison of our study with previous research.

Study	Key Findings	Our Validation
Niazkar (2020) [11]	14-day incubation period improves ANN accuracy.	We included incubation periods in preprocessing.
Devaraj et al. (2021) [15]	Stacked LSTM outperforms ARIMA and Prophet.	LSTM consistently ranked higher in our study.
Jin et al. (2023) [17]	Hybrid CNN-LSTM-ARIMA improves accuracy.	We confirm that deep learning models enhance forecasts.
Yu et al. (2021) [18]	ARIMA and FNN perform better in certain regions.	ARIMA showed stability in short-term forecasting.
Rustam et al. (2020) [20]	Longer training periods improve accuracy.	LSTM performed best with 20–30 weeks of data.
Painuli et al. (2021) [22]	ARIMA with ensemble learning enhances reliability.	Our study integrates MCDM for robust model ranking.
Kumar & Kaur (2021) [23]	SOMFTS hybrid model improves forecasting.	We used a weighted ranking approach for evaluation.
Gupta et al. (2021) [28]	Prophet excels in short-term but lacks long-term accuracy.	We confirm Prophet’s short-term success but weaker long-term results.

**Table 6:** Performance metrics with statistical significance measures.

Model	RMSE ± CI	MAE ± CI	MAPE ± CI	*p*-Value (vs. Baseline)
ARIMA	0.731 ± 0.05	0.466 ± 0.03	3.2% ± 0.5%	p=0.03
LSTM	0.676 ± 0.04	0.413 ± 0.02	2.8% ± 0.4%	p=0.02
Prophet	7.92 ± 1.10	1.68 ± 0.06	6.4% ± 1.2%	p=0.10

## Data Availability

Suggested Data Availability Statements are available in Section 3.
